# C1q reprograms innate immune memory

**DOI:** 10.3389/fimmu.2025.1515127

**Published:** 2025-05-23

**Authors:** Inge Jonkman, Maaike M. E. Jacobs, Yutaka Negishi, Julia I. P. van Heck, Vasiliki Matzaraki, Joost H. A. Martens, Marijke Baltissen, Michiel Vermeulen, Zahi A. Fayad, Abraham J. P. Teunissen, Willem J. M. Mulder, Luuk B. Hilbrands, Leo A. B. Joosten, Mihai G. Netea, Musa M. Mhlanga, Nils Rother, Raphaël Duivenvoorden

**Affiliations:** ^1^ Department of Nephrology, Research Institute for Medical Innovation, Radboud University Medical Center, Nijmegen, Netherlands; ^2^ Department of Molecular Biology, Faculty of Science, Oncode Institute, Radboud University Nijmegen, Nijmegen, Netherlands; ^3^ Department of Cell Biology, Faculty of Science, Radboud University, Nijmegen, Netherlands; ^4^ Department of Internal Medicine and Radboud Center for Infectious Diseases, Research Institute for Medical Innovation, Radboud University Medical Center, Nijmegen, Netherlands; ^5^ Biomolecular Engineering and Imaging Institute, Icahn School of Medicine at Mount Sinai, New York, NY, United States; ^6^ Diagnostic, Molecular and Interventional Radiology, Icahn School of Medicine at Mount Sinai, New York, NY, United States; ^7^ Cardiovascular Research Institute, Icahn School of Medicine at Mount Sinai, New York, NY, United States; ^8^ Icahn Genomics Institute, Icahn School of Medicine at Mount Sinai, New York, NY, United States; ^9^ Laboratory of Chemical Biology, Department of Biomedical Engineering and Institute for Complex Molecular Systems, Eindhoven University of Technology, Eindhoven, Netherlands; ^10^ Department of Medical Genetics, University of Medicine and Pharmacy, Iuliu Haţieganu, Cluj-Napoca, Romania; ^11^ Department of Immunology and Metabolism, Life and Medical Sciences Institute, University of Bonn, Bonn, Germany

**Keywords:** C1q, innate immune memory, tolerance, trained immunity, complement, immunometabolism

## Abstract

Innate immune memory, also called trained immunity, is a metabolic and epigenetically regulated process that enables innate immune cells to recalibrate their inflammatory reactivity in response to pathogenic or endogenous stimuli. In addition to its function in host defense, trained immunity contributes to diverse immune-mediated diseases. We discovered that complement component 1q (C1q) is an effective modulator of innate immune memory, potently suppressing the responsiveness of myeloid cells. We found that C1q leads to profound reprogramming of myeloid cell metabolism, particularly glycolysis, and exerts control over the transcriptional regulation of important metabolic and inflammatory genes. We corroborate our findings by identifying single-nucleotide polymorphisms close to the C1q gene that are linked to induction of trained immunity by Bacillus Calmette–Guérin (BCG) or beta-glucan in healthy peripheral blood mononuclear cells. Our results reveal an immunomodulatory role for C1q and provide evidence of a molecular interaction between the complement system and innate immune memory. These findings expand our understanding of innate immune memory.

## 1 Introduction

The interplay between the complement system and myeloid cells critically regulates innate immune responses and is essential for maintaining tissue homeostasis and orchestrating host defense against infection (1). Upon recognition of foreign invaders, complement proteins activate a cascade of enzymatic responses through several signaling mechanisms, including the classical, lectin, and alternative pathways. Subsequently, innate immune cells are recruited, which triggers inflammatory responses against microbial threats (1–4). In addition to their role in host defense, complement proteins, particularly complement component 1q (C1q), contribute to the silent clearance of apoptotic host cells, preventing chronic inflammation and autoimmunity (5–7).

C1q is a 460 kDa protein complex consisting of 18 polypeptide chains: six A chains, six B chains, and six C chains. These chains all have a globular N-terminal domain, a collagen region, and a C-terminal region. C1q is a pattern recognition molecule involved in activating the classical complement pathway. When the C1 complex, consisting of C1q, C1r, and C1s, binds to the Fc region of IgG or IgM, C1r and C1s cleave C4 into C4a and C4b and C2 into C2a and C2b. This leads to the formation of C4b2a, called classical C3 convertase, which cleaves C3 into C3a and C3b, eventually leading to the formation of the Membrane Attack Complex (MAC) (1, 8). In addition to binding to the Fc region of immunoglobulins, C1q can also bind to apoptotic cells. This does not lead to activation of the complement cascade but serves to clear apoptotic cells in an immunologically silent manner, in which C1q acts as a bridge molecule between the apoptotic cells and the phagocyte. C1q can also bind to complement receptor 1 (CR1) and C1q receptors (C1qR) on monocytes and macrophages. There are two types of C1qRs. First is the cC1qR, which binds to the collagen region of C1q, and second is the gC1qR, which binds to the globular heads of C1q (8, 9).

While the influence of complement proteins on innate immune cell activation is well established, if and how complement proteins affect innate immune memory remains unknown. It is now well documented that innate immune cells can develop immunological memory after exposure to specific pathogen-associated molecular patterns or danger-associated molecular patterns. This innate immune memory, also called trained immunity, is imprinted through metabolic and epigenetic changes and facilitates faster and stronger transcription of inflammatory genes upon subsequent stimulation (10–12). Conversely, other microbial stimuli [e.g., lipopolysaccharide (LPS)] can induce long-term suppression of the inflammatory responsiveness to secondary stimuli, a process referred to as innate immune tolerance (10–12). Thus, trained immunity and innate immune tolerance represent two contrasting responses within the spectrum of innate immune memory. Understanding how complement proteins impact innate immune memory is crucial, as this provides fundamental immunological insight into the interaction of these two co-evolved components of the innate immune system and can provide new targets for therapeutic intervention.

We investigated the effects of various complement proteins on the regulation of innate immune memory. Through a comprehensive analysis of *in-vitro* cytokine responses in human peripheral blood mononuclear cells (PBMCs), we identified a distinctive suppressive effect exerted by C1q on the responsiveness of innate immune cells. Our study integrated functional, metabolic, transcriptomic, and epigenetic data to elucidate the molecular mechanisms underpinning C1q-induced innate immune tolerance. Our findings were validated by a functional analysis of trained immunity quantitative trait loci (FTI-QTL) in the 300BCG cohort of the Human Functional Genomics Project (13), confirming the effect of C1q on innate immune memory. This reveals a previously unrecognized facet of innate immune regulation and offers crucial insights into the dynamics of innate immune memory and its implications for the pathophysiology of various diseases in which innate immunity plays a role, such as infectious diseases, cancer, atherosclerosis, autoinflammatory diseases, and organ transplantation (14–18).

## 2 Materials and methods

### 2.1 Human subjects

For *in-vitro* studies on human PBMCs, buffy coats from healthy donors were obtained from Sanquin blood bank, Geert Grooteplein Zuid 34, 6525GA Nijmegen, the Netherlands, after written consent, from which no additional details are available. Sex was not considered as a biological variable.

### 2.2 Human 300BCG cohort

The 300BCG cohort consists of healthy males and females of Western European ancestry. This study was approved by the local ethics committee (CMO region Arnhem–Nijmegen, number NL58553.091.16). Inclusion of volunteers and experiments were conducted according to the principles expressed in the Declaration of Helsinki. All volunteers gave written informed consent before any material was collected.

### 2.3 PBMC isolation

PBMCs were isolated by differential centrifugation over Ficoll-Paque (Lymphoprep, Stemcell Technologies, Cambridge, UK). Cells were washed 3 times in phosphate buffered saline (PBS), and PBMCs were resuspended in RPMI 1640 culture medium supplemented with 2 mM glutamax, 1 mM pyruvate, and penicillin/streptomycin (all from Thermo Fisher Scientific, Breda, The Netherlands) and counted on a Casy cell counter.

### 2.4 Complement proteins

Complement proteins C3, C3a, C3b, C4, C4a, C4b, and C5a were purchased from Complement Technology, Bio-connect, Huissen, The Netherlands and MBL from R&D systems, Dublin, Ireland. Endotoxin concentration in C1q was determined using the Pierce Chromogenic Endotoxin Quant kit and was 1.5 EU/ml (equal to around 0.15–0.3 ng/ml LPS) for the highest used concentration (300 g/ml).

### 2.5 Trained immunity assays

**Figure 1:**
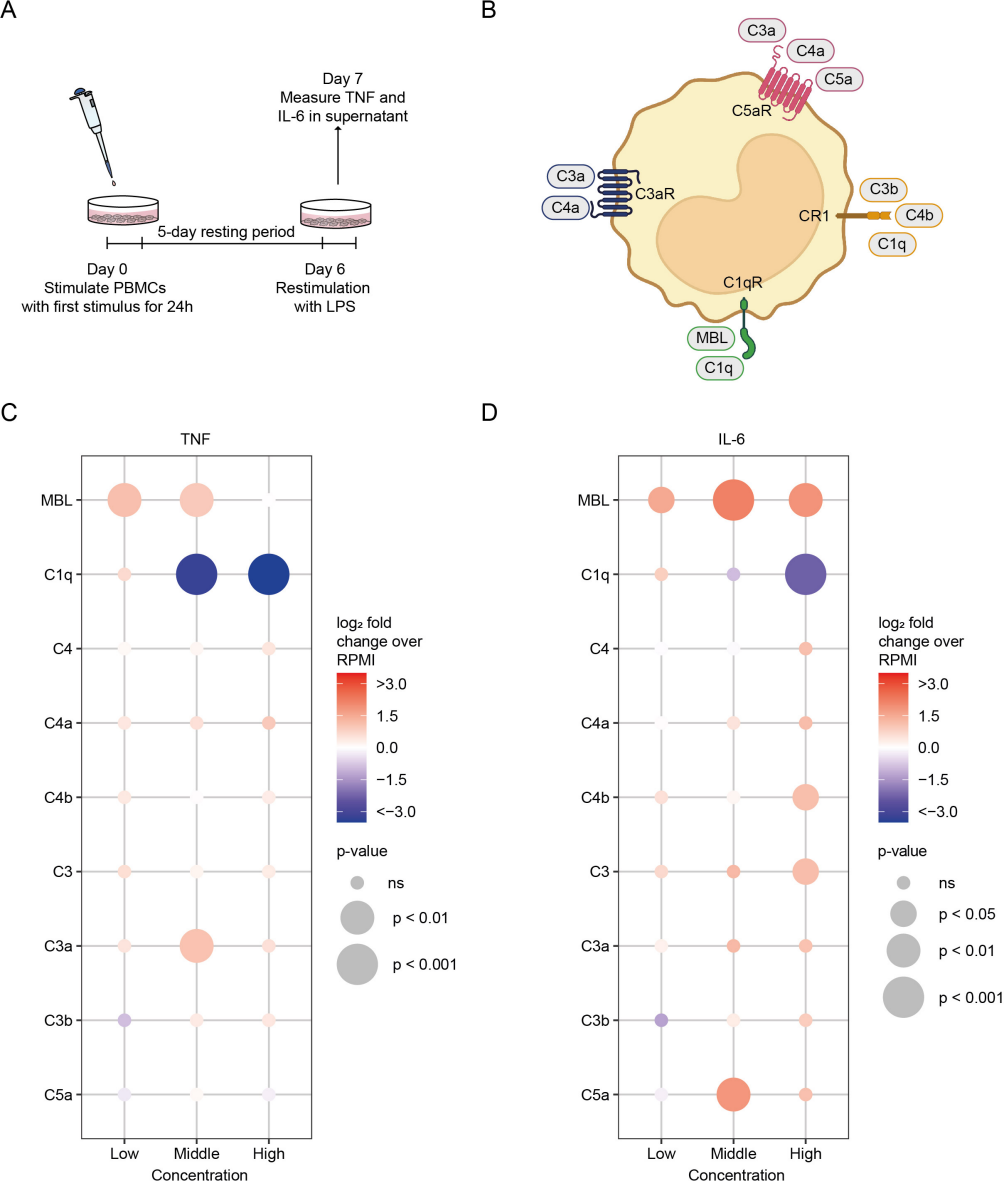
The effects of complement proteins on innate immune memory responses. **(A)** Schematic representation of the innate immune memory response assay. **(B)** Schematic simplified representation of the interaction of complement proteins with monocytes and macrophages. Factors in bold were tested for their ability to modulate innate immune memory responses. **(C, D)** PBMCs were stimulated for 24h with complement factors in low, medium, and high concentrations, after which the stimulus was washed away. After a 5-day resting period, cells were restimulated with LPS (10 ng/ml) for 24h and cytokine production was measured in the supernatant by ELISA (*n* = 6 donors). Data are expressed as log_2_ fold change compared to untrained (RPMI) PBMCs. p-values were calculated using an unpaired *t*-test. ns: not significant. Concentrations used for low, medium and high groups are: MBL 500 ng/ml, 10 and 20 µg/ml; C1q 50, 150 and 300 µg/ml; C4 1, 10, 50 µg/ml; C4a 0.5, 2, 5 µg/ml; C4b 0.5, 2, 5 µg/ml; C3 100 ng/ml, 1, 10 µg/ml; C3a 100, 500 ng/ml, 1 µg/ml; C3b 100, 500 ng/ml, 1 µg/ml; C5a 100, 250, 500 ng/ml.

Human PBMCs were plated in 96-well flat-bottom plates, with 500,000 cells per well. Cells were let to adhere for one hour at 37°C. After 1h, cells were washed –3 times with PBS. After washing, PBMCs were incubated with culture medium only as a negative control, heat-killed Candida albicans (HKCA), 10^5^ cells/ml (InvivoGen, Toulouse, France), Bacillus Calmette–Guérin (BCG) vaccine (5 μg/ml, BCG strain Bulgaria, Intervax) that acted as a positive control, or a complement factor for 24h at 37°C. To assess the effect of efferocytosis inhibition, cells were co-incubated with BMS794833 (10 µM, Bioconnect, Huissen, The Netherlands). After 24h, cells were washed and rested for 5 days in culture medium containing 10% FBS. After the resting period, cells were stimulated with either RPMI culture medium as a negative control or 10 ng/ml LPS (InvivoGen, Toulouse, France) for 24h. After 24h supernatant was collected. For *in vitro* cytokine production in **Figure 1**; **Supplementary Figures S1–S3**, two technical replicates were performed for each donor.

### 2.6 Lactate dehydrogenase measurements to assess cellular toxicity

LDH concentration was measured in supernatants of PBMCs after 24-h incubation with complement factors using the CyQuant LDH Cytotoxicity Assay (Thermo Fisher Scientific, Breda, The Netherlands). LDH concentration was calculated as a percentage of maximal possible LDH concentration in completely lysed cells according to the formula:


$$LDH\left(\%ofmax\right)=\left(\frac{CompoundinducedLDH-SpontaneousLDH}{MaximumLDH-SpontaneousLDH}\right)x100$$

### 2.7 Annexin V/PI measurements to assess cellular toxicity

Annexin V/PI measurement was performed on PBMCs after 24-h incubation with a range of C1q concentrations using the FITC Annexin V Apoptosis detection kit (BioLegend, Amsterdam, The Netherlands) according to manufacturer’s instruction. Cells were acquired with an ACEC Novocyte 300 (Agilent, Santa Clara, CA, US).

### 2.8 Cytokine/protein measurements

Cytokine production was measured in supernatants using commercial ELISA kits for human TNF, IL-6, and IL-1β (R&D systems, Dublin, Ireland) according to manufacturer’s instruction.

### 2.9 Monocyte isolation

Monocytes were isolated from PBMCs by negative MACS isolation using MojoSort Human Pan Monocyte Isolation Kit (Biolegend, Amsterdam, The Netherlands). Briefly, stimulated PBMCs were washed with PBS and incubated in Versene solution (0.48 mM EDTA) for 30 min at 37°C. Cells were scraped, counted, and resuspended in MACS buffer (PBS with 0.5% BSA and 2 mM EDTA). Monocyte isolation was performed according to manufacturer’s instructions.

### 2.10 RNA isolation and sequencing for transcriptomic analysis

**Figure 2:**
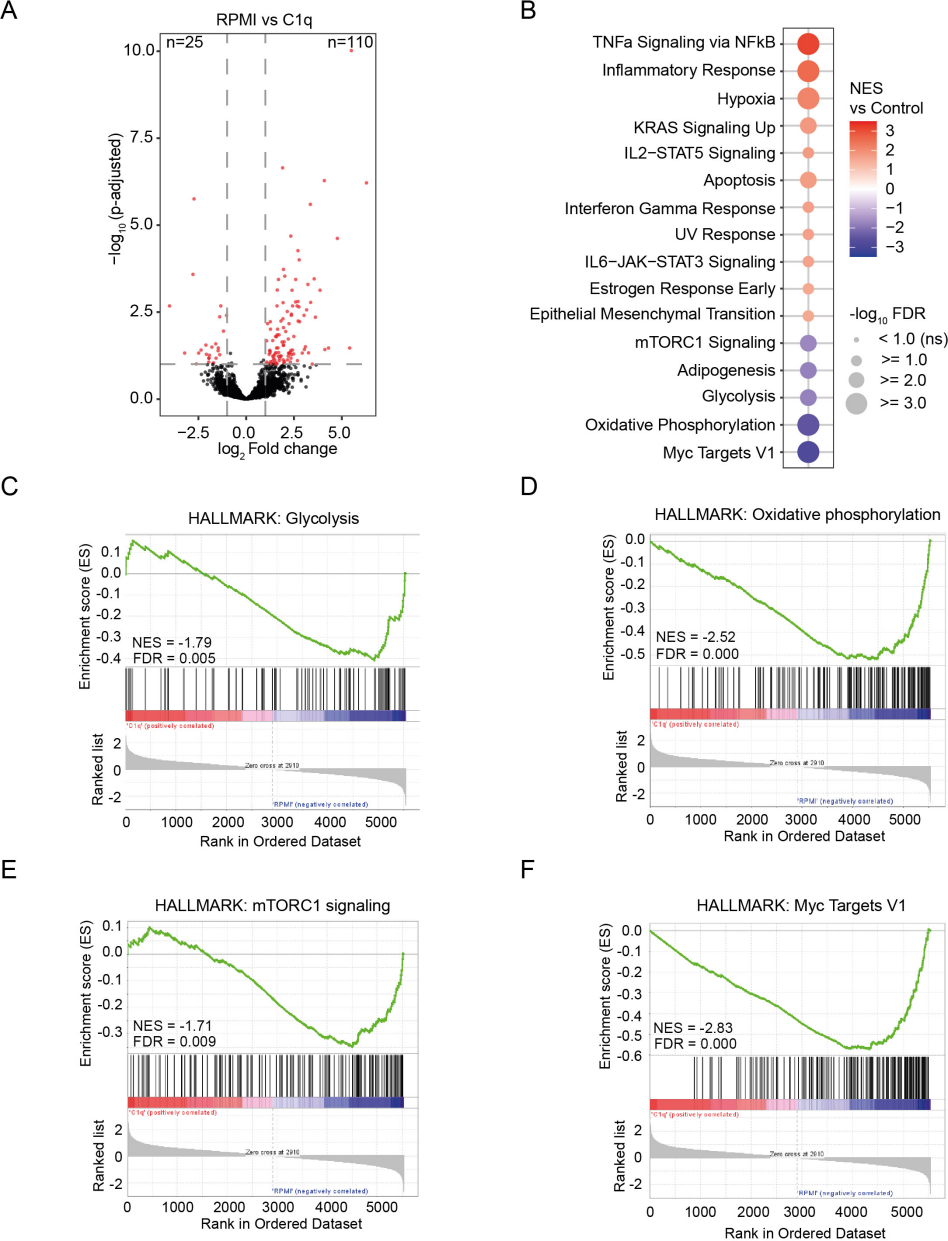
C1q stimulation affects the transcriptome of monocytes. **(A)** Volcano plot representing RNA-seq data of monocytes stimulated with C1q (300 µg/ml) compared to control (RPMI). Significantly up-and downregulated genes are reported as red dots. Non-DEGs are presented as black dots. The cutoff for significance was *p*-adjusted< 0.1, fold change > 2, (*n* = 3 donors). **(B)** Significantly altered gene sets of the HALLMARK database in monocytes stimulated with C1q (300 µg/ml) versus control (RPMI). FDR< 0.1. **(C–F)** Gene set enrichment analysis of C1q (300 µg/ml) versus control (RPMI) dataset for the HALLMARK gene sets “Glycolysis” **(C)**, “Oxidative phosphorylation” **(D)**, “mTORC1 signaling” **(E)**, and “Myc Targets V1” **(F)**. NES, normalized enrichment score; FDR, false discovery rate.

Human PBMCs were treated with 300 µg/ml C1q or RPMI culture medium containing 10% FBS for 24h at 37°C. After 24h, monocytes were isolated from PBMCs by negative MACS isolation using the MojoSort Human Pan Monocyte Isolation Kit (BioLegend, Amsterdam, The Netherlands). RNA was isolated from 1 × 10^6^ monocytes using the RNeasy Mini kit (Qiagen, Hilden, Germany) including DNAse I digestion according to manufacturer’s instructions. RNA bulk sequencing was performed by Single Cell Discoveries (Utrecht, the Netherlands) with a sequencing depth of 20 million reads/sample. Library preparation was performed according to the CEL-seq2 protocol (19). Sequencing was performed on a Nextseq500 (Illumina, Cambridge, UK). RNA-sequencing experiment (**Figure 2**; **Supplementary Figure S4**) was performed in a single technical replicate for each donor.

**Figure 3:**
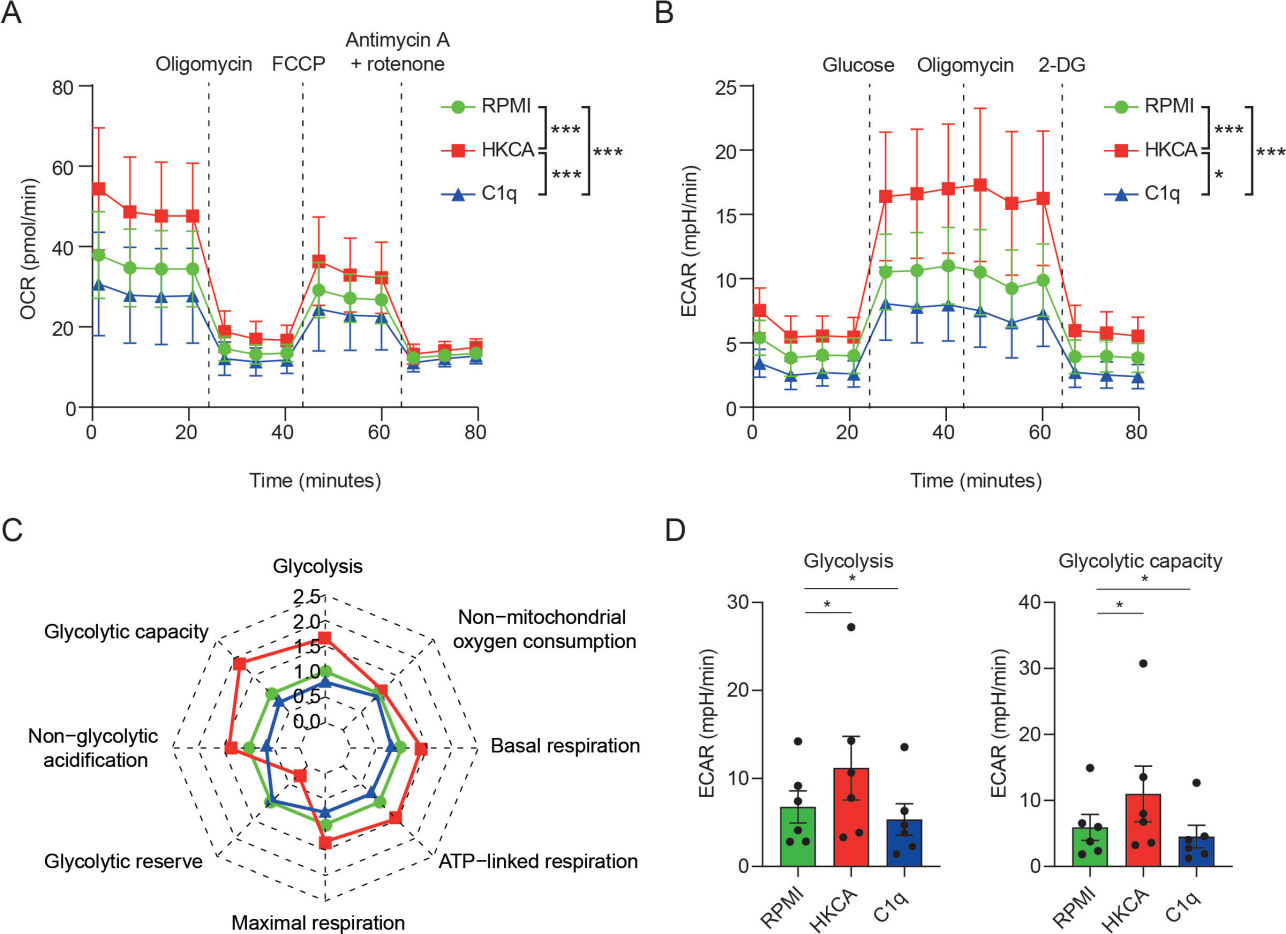
C1q durably alters the metabolism of human primary monocytes. **(A–D)** PBMCs were stimulated for 24h with C1q (300 µg/ml), or with HKCA or RPMI as positive and negative controls, respectively. After a 5-day resting period, the cells’ metabolic activity was assessed by Seahorse analysis (*n* = 6 donors). Metabolic parameters **(C, D)** were calculated from the oxygen consumption rate (OCR) **(A)**, or the extracellular acidification rate (ECAR) **(B, A)** OCR upon injection of oligomycin, carbonyl cyanide-4-(trifluoromethoxy) phenylhydrazone) (FCCP) and antimycin A + rotenone at indicated time points, in PBMCs stimulated with C1q (300 µg/ml), HKCA, or RPMI measured 6 days after treatment using Seahorse technology (*n* = 6 donors). Mean ± SEM. **(B)** Extracellular acidification rate (ECAR) upon injection of glucose, oligomycin, and 2-deoxyglucose (2-DG) at indicated time points, in PBMCs stimulated with C1q (300 µg/ml), HKCA, or RPMI measured 6 days after treatment using Seahorse technology (*n* = 6 donors). Mean ± SEM. **(C)** Spider plot of oxidative phosphorylation and glycolysis parameters as analyzed with Seahorse technology 6 days after stimulation in PBMCs stimulated with C1q (300 µg/ml), HKCA, or RPMI measured 6 days after treatment using Seahorse technology (*n* = 6 donors). **(D)** Glycolysis rate, and glycolytic capacity analyzed with Seahorse technology in PBMCs stimulated with C1q (300 µg/ml), HKCA, or RPMI measured 6 days after treatment using Seahorse technology (*n* = 6 donors). Data are represented as mean ± SEM **(A, B, D)** and mean fold change compared to RPMI control (RPMI) **(C)**. Two-way repeated measures ANOVA for **(A)** *F (*24,120) = 5.712, *p* = 4.2 × 10^−11^ and **(B)** *F (*24,120) = 4.352, *p* = 3.44 × 10^−8^; Bonferroni adjusted *p*-values of paired *t*-test between condition are indicated as *p< 0.05, ***p< 0.001. *p*-values in **(D)** were calculated using a one-tailed paired *t*-test.

**Figure 4:**
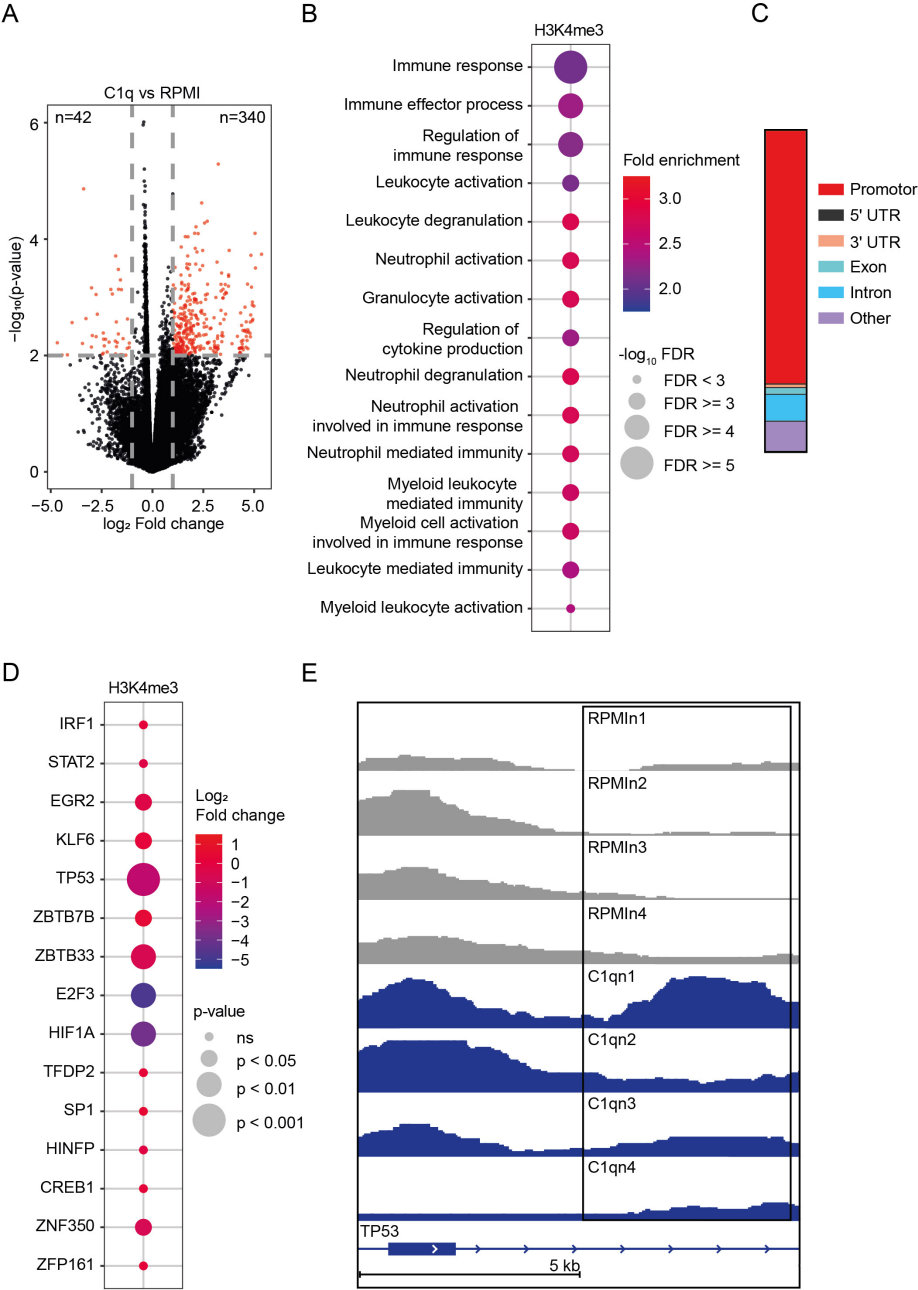
Epigenetic profile of C1q-tolerized monocytes. **(A)** Volcano plot of genomic regions with altered abundance of H3K4me3 marks (fold change > 2 or< 0.5, *p*-value< 0.01) in monocytes stimulated for 24h with C1q (300 µg/ml) versus unstimulated monocytes (RPMI), 6 days after stimulation (*n* = 4 donors). **(B)** Top 15 Gene Ontology (GO) Biological Processes associated with genomic regions showing an altered abundance of H3K4me3 marks in monocytes stimulated for 24h with C1q (300 µg/ml) versus unstimulated monocytes (RPMI), as determined by ChIP-seq 6 days after stimulation (*n* = 4 donors). **(C)** Genomic annotations of H3K4me3 peaks (*p*< 0.01) **(D)** Transcription factors related to innate immune tolerance showing an altered abundance of H3K4me3 marks in monocytes stimulated for 24h with C1q (300 µg/ml) versus unstimulated monocytes (RPMI), as determined by ChIP-seq 6 days after stimulation (*n* = 4 donors). **(E)** H3K4me3 signal at TP53 as visualized in the Integrative Genomics Viewer. RPMI samples (*n* = 4 donors) compared to C1q (300 µg/ml) samples (*n* = 4 donors).

### 2.11 Seahorse experiments

Human PBMCs were treated with 300 µg/ml C1q, 10^5^ cells/ml HKCA (InvivoGen, Toulouse, France), or RPMI culture medium containing 10% FBS for 24h at 37°C. After 24h, cells were washed and rested for 5 days in culture medium containing 10% FBS. After the 5-day resting period, cells were washed with PBS and incubated in Versene solution in PBS for 30 min at 37°C. Cells were scraped, counted, and seeded in a density of 100,000 cells per well in quintuples in XF96 microplates (Agilent Technologies, Santa Clara, CA, US). Cells were allowed to adhere for 1hour at 37°C. After this, cells were kept in a CO_2_-free incubator (37°C) for 45–60 min, followed by measurements of oxygen consumption and extracellular acidification. These measurements were performed at 37°C using a Seahorse XF96 Extracellular Flux Analyzer (Agilent Technologies, Santa Clara, CA, US). The Seahorse XF Cell Glyco Stress Test was performed to determine the dynamics of the glycolytic rate based on the extracellular acidification rate (ECAR). The cells were treated with glucose (11 mM), oligomycin (1 μM; Sigma-Aldrich, Amsterdam, The Netherlands), and 2-deoxy-d-glucose (22 mM; Sigma-Aldrich, Amsterdam, The Netherlands), respectively, at time points indicated in **Figure 3B**. To determine the dynamics in mitochondrial oxygen consumption rate (OCR), the Seahorse XF Cell Mito Stress Test was performed. During this test, cells were treated with oligomycin (1 μM; Sigma-Aldrich, Amsterdam, The Netherlands), carbonyl cyanide-4-(trifluoromethoxy) phenylhydrazone (FCCP; 1 μM; Sigma-Aldrich, Amsterdam, The Netherlands), and a combination of rotenone (1.25 μM; Sigma-Aldrich, Amsterdam, The Netherlands) and antimycin A (2.5 μM; Sigma-Aldrich, Amsterdam, The Netherlands) at time points indicated in **Figure 3A**. For seahorse metabolic experiments in **Figure 3**; **Supplementary Figure S5**, six technical replicates were performed for each donor.

### 2.12 Chromatin immunoprecipitation

Human PBMCs were treated with 300 µg/ml C1q or RPMI culture medium containing 10% FBS for 24h at 37°C. After 24h cells were washed and cultured in RPMI culture medium for 5 days. On day 6, monocytes were isolated from PBMCs by negative MACS isolation using MojoSort Human Pan Monocyte Isolation Kit (BioLegend). Monocytes were resuspended in RPMI culture medium and fixed using formaldehyde (1% final concentration, Sigma-Aldrich, Amsterdam, The Netherlands) for 10 min at room temperature. Unreacted formaldehyde was quenched with 125 mM glycine and incubated for 5 min at room temperature. Cells were washed twice in PBS containing protease inhibitor cocktail (Roche, Woerden, The Netherlands) and 1 mM PMSF (Roche, Woerden, The Netherlands) and subsequently snap frozen in liquid nitrogen. Cell pellets were stored at −80°C for further use. Cells were sonicated at a concentration of 15 million cells/ml using a Bioruptor pico sonicator (Diagenode, Liege, Belgium; 10–20 cycles, 30s on, 30s off), at 4°C. Immunoprecipitation was performed using the MagnaChIP kit (Merck Millipore, Darmstad, Germany) according to the manufacturer’s instructions. In short, 500,000 cells were incubated overnight with 1 mg H3K4me3 (Diagenode, Liege, Belgium) and protein A magnetic beads at 4°C. Beads and chromatin/antibody mixture were washed 4 times for 5 min at 4°C. After washing, chromatin was eluted and proteins were degraded using proteinase K. DNA was purified using spin columns and eluted in millliQ. The ChIP-sequencing experiment (**Figure 4**) was performed in a single technical replicate for each donor.

### 2.13 Library preparation and sequencing of ChIP samples

ChIP-seq libraries were prepared using the Kapa Hyper Prep Kit according to manufacturer’s protocol, with the following modifications. 2.5 ml of the NEXTflex adaptor stock (600 nM, Bio Scientific, Austin, TX, US) was used for adaptor ligation of each sample. Libraries were amplified with 12–15 PCR cycles followed by a double post-amplification clean-up used to ensure proper removal of adapters. Samples were analyzed for purity using a high-sensitivity DNA Chip on a Bioanalyzer 2100 system (Agilent, Santa Clara, CA, US). Libraries were paired-end sequenced to a read length of 50 bp on an Illumina, Cambridge, UK NextSeq500.

### 2.14 *In vitro* training in the 300BCG cohort

**Figure 5:**
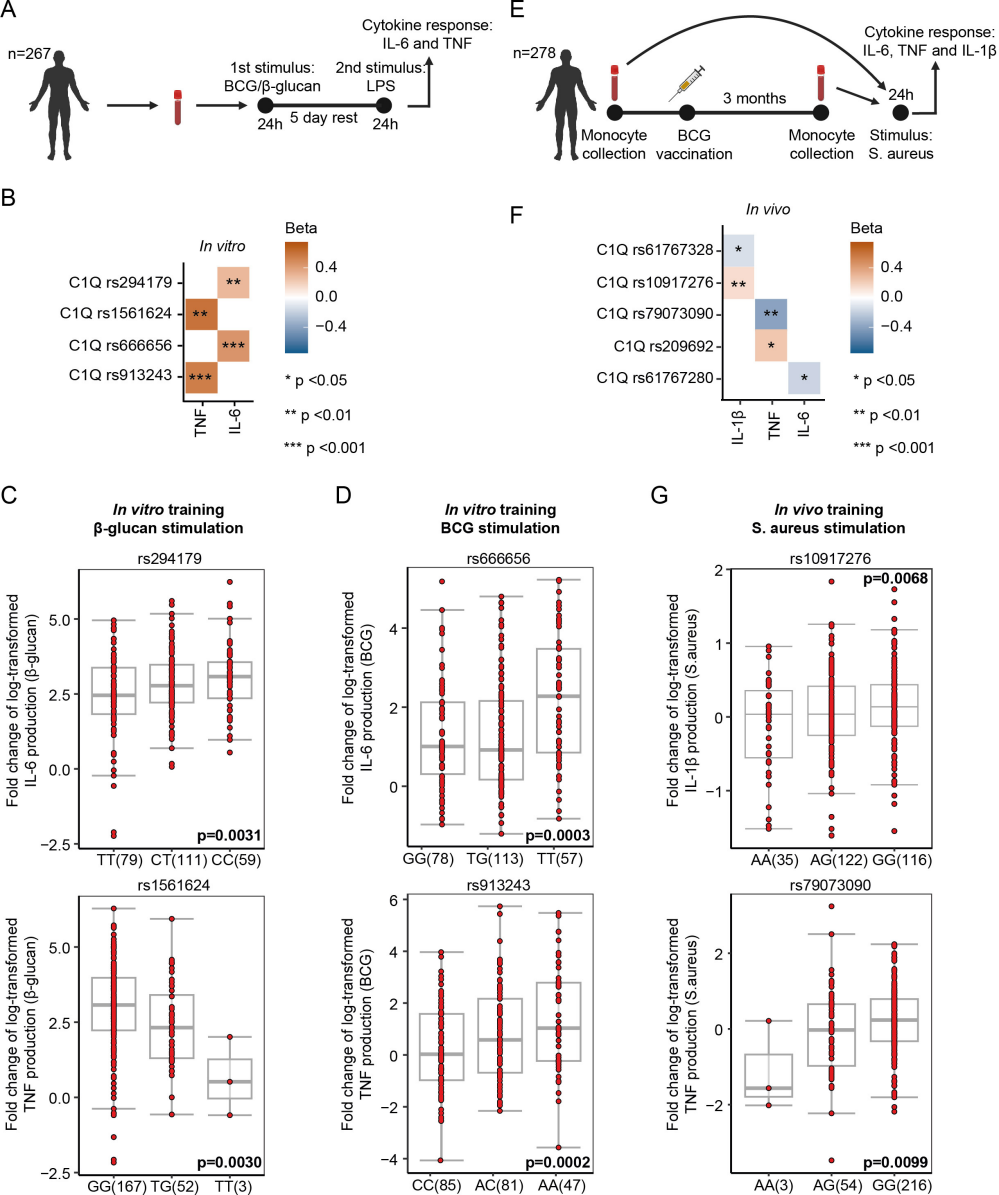
Single nucleotide polymorphisms in the proximity of genes encoding C1q components associate with innate immune memory responses. **(A)** Schematic representation of the *in-vitro* training experiments followed by QTL analysis using single nucleotide polymorphism (SNP) genotypes of volunteers of the 300BCG cohort. **(B)** Heatmap of SNPs that show a significant association with the capacity of β-glucan/BCG-induced trained immunity (*n* = 267 donors). Beta shows the direction of the effect (positive = SNP increases the fold change of cytokine production upon training, and negative = SNP decreases the fold change of cytokine production). **(C)** Associations of SNPs rs294179, rs1561624 near C1q with the cytokine responses after 24h restimulation with LPS (10 ng/ml), 5 days after β-glucan training (*n* = 267 donors). Boxplots show the genotype stratified fold changes in IL-6 and TNF responses for SNPs rs294179 and rs1561624. **(D)** Associations of SNPs rs666656, and rs913243 near C1q with the cytokine responses after 24h restimulation with LPS (10 ng/ml), 5 days after BCG training (*n* = 267 donors). Boxplots show the genotype stratified fold changes in IL-6 and TNF responses for SNPs rs666656 and rs913243. **(E)** Schematic representation of the *in-vivo* training experiments followed by QTL analysis using SNP genotypes of volunteers of the 300BCG cohort. **(F)** Heatmap of SNPs that show a significant association with the capacity of the BCG vaccine-induced trained immunity (*n* = 278 donors). Beta shows the direction of the effect (positive = SNP increases the fold change of cytokine production upon training, negative = SNP decreases the fold change of cytokine production) **(G)** Associations of SNPs rs10917276 and rs79073090 near C1q with the cytokine responses after vaccination with Bacillus Calmette–Guérin (BCG) vaccine (*n* = 278 donors). Boxplots show the genotype stratified fold changes in IL-1β and TNF responses for SNPs rs10917276 and rs79073090, respectively.

*In-vitro* BCG/β-glucan–trained immunity assays were performed on PBMCs of 267 healthy individuals of the 300BCG cohort. Both cytokine and genotype data were available for these patients. PBMCs were plated in 96-well flat-bottom plates, with 500,000 cells per well. They were stimulated with BCG (5 μg/ml, BCG strain Bulgaria, Intervax) or β-glucan (provided by Professor David Williams, College of Medicine, Johnson City, USA) or RPMI (control) for 24h, washed with PBS, and rested for 5 days in RPMI culture medium. After the resting period, cells were restimulated with 10 ng/ml LPS (O55:B5, Sigma-Aldrich, Amsterdam, The Netherlands) for 24h and supernatants were collected. TNF and IL-6 concentrations were measured, and fold changes between trained and control were calculated. DNA samples of these individuals were genotyped using the commercially available Single Nucleotide Polymorphism chip (SNP chip), Infinium Global Screening Array MD version 1.0 from Illumina, Cambridge, UK. Opticall 0.7.0 with default settings was used for genotype calling. Standard quality control per SNP and sample was performed as previously described (20). For *in vitro* cytokine production in **Figure 5**, two technical replicates were performed for each donor.

### 2.15 *In vivo* training in the 300BCG cohort

Individuals from the 300BCG cohort were vaccinated with 0.1 ml of BCG (BCG vaccine strain Bulgaria; Intervax, Canada). PBMCs were isolated and stimulated *ex vivo* with 5 × 10^6^ CFU/ml heat-killed *Staphylococcus aureus* before vaccination and 90 days after vaccination. IL-1β, TNF, and IL-6 cytokine concentrations were measured after 24h in the supernatants, and the fold change (after vaccination over baseline) in cytokine production was used as readout for the trained immunity response. Both cytokine and genotype data were available for these patients. DNA samples of these individuals were genotyped using the commercially available SNP chip, Infinium Global Screening Array MD version 1.0 from Illumina, Cambridge, UK. Opticall 0.7.0 with default settings was used for genotype calling. Standard quality control per SNP and sample was performed as previously described (20).

### 2.16 QTL mapping

The fold changes of cytokine production were log-transformed and mapped to genotype data using a linear regression model with age and sex as covariates. The R-package Matrix-eQTL was used for cytokine QTL mapping.

### 2.17 Statistical analysis

#### 2.17.1 *In vitro* experiments data analysis

For *in-vitro* training experiments, data is shown as mean ± SEM, and significance is tested with an unpaired *t*-test. Data were analyzed using Graphpad Prism 9.5.0. A *p*-value of 0.05 was considered to be statistically significant.

#### 2.17.2 Seahorse metabolic parameters analysis

The metabolic parameters depicted in **Figures 3C, D** and **Supplementary Figure S5** were calculated from the OCR and ECAR as follows. The basal respiration was calculated as the average OCR before adding oligomycin. ATP-linked respiration was calculated as the average OCR of time points 1–4 subtracted by the average OCR of time points 5–7. The maximal respiration was calculated as the average OCR of time points 8–10 subtracted by the average OCR of time points 11–13. The spare respiratory capacity was calculated as the average OCR of time points 8–10 subtracted by the average OCR of time points 1–4. Non-mitochondrial oxygen consumption was calculated as the average OCR of time points 11–13. Glycolysis was calculated as the average ECAR of time points 5–7 subtracted by the average ECAR of time points 1–4. The glycolytic capacity was calculated as the average ECAR of time points 8–10 subtracted by the average ECAR of time points 1–4. Non-glycolytic acidification was calculated as the average ECAR of time points 1–4. Glycolytic reserve was calculated as the average ECAR of time points 8–10 subtracted by the average ECAR of time points 5–7. Metabolic parameters were tested for significance using an unpaired *t*-test.

#### 2.17.3 RNA-seq data analysis

Differential gene expression analysis was performed on counts of three samples per treatment group using the DESeq2 package in Rstudio (21). Samples were paired for donors. Differentially expressed genes (DEGs) were defined as fold change (FC)< 0.5 or FC > 2 and FDR< 0.1 (22).

#### 2.17.4 Gene set enrichment analysis

Gene set enrichment analyses (GSEA) were performed on normalized counts of three samples per treatment group using GSEA software v4.1.0 provided by the Broad Institute (23). GSEA was performed using the gene sets from the HALLMARK database of the Molecular Signature database (MSigDB) (24, 25). Analyses were conducted with 1,000 gene set permutations and with the following settings: metric for ranking genes: Signal2Noise; Remap/Collapse to gene symbols: collapse; enrichment statistic: weighted; normalization mode: meandiv. For each gene set, a normalized enrichment score (NES) was calculated. Gene sets for which FDR< 0.1 were considered to be enriched.

#### 2.17.5 ChIP-seq data analysis

ChIP-sequencing data was aligned to human genome hg38 with BWA (26). Samtools was used to filter reads with a quality score lower than 20, and PCR duplicates were removed with Picard (27). Peaks were identified with MACS 2.2.6 in paired-end mode and “call-summits” enabled at a false discovery rate (FDR) of 0.01 (28). A union of all identified peaks was generated with BEDTools, which was used to count reads per peak in each sample (29). Count matrix data was loaded to the R programming language, and differential H3K4me3 loci were identified by using the R package edgeR (30). The confident H3K4me3 signals [counts per million (CPM) >1 in more than two samples] were used for downstream analysis, and trimmed mean of M-values (TMM) normalization was performed. Then, it was adjusted for differences between donors by an additive linear model with donor as the blocking factor, and the dispersion was estimated. The differential H3K4me3 signals were identified by fitting negative binomial generalized linear models. The H3K4me3 signals with *p*-value< 0.01 were subjected to gene ontology analysis. The annotation of H3K4me3 signals was performed with R packages ChIPSeeker (31) and clusterProfiler (32). We used GREAT to identify associated gene ontologies.

## 3 Results

### 3.1 Complement factors modulate innate immune memory

To systematically investigate the impact of complement proteins on innate immune memory, we employed a well-established *in-vitro* protocol using primary human PBMCs. We chose to use PBMCs for these protocols since the interaction of monocytes with other immune cells (e.g., T cells) is important in the induction of trained immunity (33, 34). At the end of the protocol, mainly adherent monocytes remain in the culture, as non-adherent cells are washed away by subsequent washes. The protocol comprises a 24-h initial stimulation, a 5-day resting period, and a subsequent 24-h restimulation with LPS. HKCA served as a trained immunity-inducing positive control, while RPMI culture medium served as a negative control. Trained immunity and tolerance responses were assessed by measuring interleukin-6 (IL-6) and tumor necrosis factor (TNF) concentrations in the supernatant following LPS restimulation (**Figure 1A**). The ratio between cytokine production of cells incubated with a particular stimulus and the production by (negative) control cells served as a biomarker of trained immunity induction.

We focused on nine complement proteins selected to encompass key components of the complement system known to engage receptors on monocytes and macrophages (**Figure 1B**). Mannose-binding lectin (MBL) and C1q activate the classical and lectin pathways, respectively. We also selected C4 and its cleavage products C4a and C4b, C3 and its cleavage products C3a and C3b, and C5a. Notably, C3a, C4a, and C5a function as anaphylatoxins mediating chemotaxis and inflammation, while C3b and C4b act as opsonins crucial for downstream complement cascade activation. All complement factors were tested at non-toxic concentrations, as confirmed by lactate dehydrogenase (LDH) assays and AnnexinV/PI staining (LDH cell death<20%, **Supplementary Figure S1**). Compared to the RPMI negative control, the HKCA positive control induced 4.50-fold TNF and 4.52-fold IL-6 production in response to LPS restimulation (**Supplementary Figure S2**). Stimulation of PBMCs with the various complement proteins resulted in distinctly different functional responses. Stimulation with low and medium concentrations of MBL enhanced TNF (2.18- and 1.93-fold change, respectively), and all MBL concentrations enhanced IL-6 production (2.88-, 4.62-, and 3.72-fold change, respectively), following LPS restimulation. Relatively high concentrations of C3 and C4b and the medium concentration of C5a amplified IL-6 production (2.23-, 2.20-, and 3.60-fold change, respectively). The medium concentration of C3a similarly increased TNF production (2.09-fold change) after LPS restimulation. Conversely, stimulation of PBMCs with medium and high concentrations of C1q consistently exhibited a potent suppressive effect on TNF (0.10- and 0.003-fold change), and high C1q concentrations similarly suppressed IL-6 production (0.03-fold change) following LPS restimulation (**Figures 1C, D**; **Supplementary Figure S2**). Of note, C1q was also able to suppress cytokine production induced by trained immunity with HKCA or BCG (**Supplementary Figures S3A, B**). C1q’s suppressing effect cannot be attributed to an increased rate of efferocytosis, as blocking efferocytosis did not diminish C1q-induced cytokine reduction (**Supplementary Figure S3C**).

Our results unveil the intrinsic capacity of complement factors to modulate innate immune memory responses. Notably, our data posit that MBL, C3, C3a, C4b, and C5a can induce trained immunity, while C1q emerges as the sole factor exerting an inhibitory effect. This intriguing observation prompts further investigation to decipher the molecular mechanisms underlying this phenomenon. Because C1q stimulation in physiological concentrations had the most robust and consistent effect of all complement proteins and because C1q-induced innate immune tolerance may play a role in the pathophysiology of immune-mediated disorders, we decided to focus on C1q (35, 36).

### 3.2 C1q suppresses genes related to cell metabolism

To unravel how C1q induces innate immune tolerance, we studied its impact on gene transcription in human primary monocytes. We stimulated PBMCs for 24h with C1q or RPMI control and isolated monocytes for RNA-sequencing analyses. Notably, stimulation with C1q revealed a distinct transcriptional profile, leading to the identification of 135 DEGs (FC > 2 or< −2, FDR< 0.1; **Figure 2A**; **Supplementary Figure S4A**). Employing Gene set enrichment analysis (GSEA) using the Molecular Signatures Database (MSigDB) Hallmark gene set collection, we uncovered a notable enrichment of genes associated with inflammation in C1q-stimulated cells (**Figure 2B**; **Supplementary Figures S4B, C**). Interestingly, we found a downregulation of gene sets linked to glycolysis and oxidative phosphorylation (**Figures 2B–D**), indicating a suppressive effect of C1q on cell metabolism. Furthermore, we observed a dampening of Mammalian Target of Rapamycin Complex 1 (mTORC1) signaling and myelocytomatosis oncogene (MYC) target 1 gene sets (**Figures 2B, E, F**). mTORC1 signaling plays a pivotal role in glycolytic induction in trained monocytes, and MYC is a transcription factor influencing the metabolic reprogramming of innate immune cells (37, 38). These data suggest that C1q-induced innate immune tolerance is characterized by immunometabolic changes.

### 3.3 C1q durably alters the metabolism of human primary monocytes

To further clarify the immunometabolic mechanisms underlying C1q-induced changes in innate immune memory, we examined the effect of C1q on the metabolic activity of human monocytes (11). PBMCs were stimulated for 24h with C1q, or with HKCA as a positive control, or with RPMI as a negative control. Following a 5-day resting period, we conducted a comprehensive analysis of cellular metabolic activity using Seahorse’s extracellular flux analysis. Glycolytic parameters were assessed by measuring changes in ECAR in response to glucose and oligomycin. Oxidative phosphorylation parameters were assessed by monitoring changes in OCR in response to oligomycin, carbonyl cyanide 4-(trifluoromethoxy)phenylhydrazone (FCCP), and rotenone/antimycin A.

C1q notably altered cellular metabolism up to at least 5 days after stimulation. Specifically, we found that C1q durably reduced monocytes’ glycolysis and glycolytic capacity compared to RPMI, as evidenced by the changes in ECAR (**Figures 3A–D**; **Supplementary Figure S5**). This finding corroborates our transcriptional data obtained 24h after stimulation and highlights the role of metabolic changes in C1q-induced innate immune memory, and provides the long-term capacity of innate immune cells to respond to stimulation.

### 3.4 C1q induces histone modifications in immune-regulating genes

After illuminating the effect of C1q on the transcriptome and metabolism, we investigated its impact on the monocytes’ epigenome. For this purpose, we incubated PBMCs with C1q or RPMI as detailed above and, on day 6, isolated monocytes for a comprehensive genome-wide analysis of H3K4me3 through chromatin immunoprecipitation sequencing (ChIP-seq).

We found a global increase in H3K4me3 marks, with 340 genes showing increased and 42 genes showing decreased levels (log_2_ FC > 1, *p*< 0.01) in monocytes stimulated with C1q compared to RPMI (**Figure 4A**). Pathway analyses of genes proximal to the differentially regulated H3K4me3 peaks, conducted using the Genomic Regions Enrichment of Annotations Tool (GREAT), indicated significant enrichment in pathways related to immune regulation (**Figure 4B**). Most H3K4me3 peaks were located in promoters and introns (**Figure 4C**). Given that C1q induces innate immune tolerance, similar to LPS, we investigated whether C1q-induced H3K4me3 modifications are similar to those established by LPS. Previous work by Novakovic et al. described the epigenetic landscape of LPS-tolerized monocytes. They assessed H3K27ac in purified monocytes after LPS tolerization and 5 days of rest. Motif analysis in tolerized promoter and enhancer regions identified transcription factors important in tolerization (39). We found an increase in H3K4me3 marks in the transcription factors identified by Novakovic, particularly pronounced for TP53, ZBTB33, E2F3, and HIF1A (**Figures 4D, E**). The elevated H3K4me3 levels of these transcription factors are associated with an open chromatin state and active gene transcription, indicating that these genes are upregulated in response to C1q stimulation. Interestingly, in the study by Novakovic et al., the most LPS-tolerized gene promoters were enriched with HIF1A and TP53 motifs, and they identified p53 as a top tolerized pathway, which is also reflected in our analysis (39).

Together, our findings indicate that C1q-induced innate immune tolerance is characterized by significant epigenetic reprogramming in monocytes, consistent with previously described effects of LPS-tolerized monocytes.

### 3.5 Single-nucleotide polymorphisms around the genes encoding C1q components associate with innate immune memory responses

To further explore the regulatory role of C1q in innate immune memory, we conducted FTI-QTL analyses using genotype data and cytokine measurements of human primary monocytes isolated from blood obtained from 267 participants (44% males) in the 300BCG cohort of the Human Functional Genomics Project (**Figure 5A**) (13). The cohort comprised individuals aged between 18 and 71 years. Genotyping of DNA samples from all participants was performed to identify single-nucleotide polymorphisms (SNPs). Two distinct FTI-QTL analyses were performed, the first involving *ex-vivo* training of monocytes and the second using an *in-vivo* training stimulus.

In the initial FTI-QTL analysis, monocytes were *in vitro* stimulated with BCG or β-glucan to induce trained immunity. After a five-day resting period, cells were restimulated with LPS for 24h, and TNF and IL-6 cytokine concentrations were quantified in the supernatant (**Figure 5A**). Our genomic analyses revealed four SNPs (within a 250-kb window) in the proximity of the genes encoding the three peptide chains of C1q (C1QA, C1QB, and C1QC, located on chromosome 1), exhibiting associations with the capacity of β-glucan and BCG to induce trained immunity. For cells trained with β-glucan, SNPs rs294179 and rs1561624 were linked to the IL-6 (*n* = 249, *p* = 0.0031) and TNF (*n* = 222, *p* = 0.0030) trained immunity response, respectively. For cells trained with BCG, SNPs rs666656 and rs913243 were linked to the IL-6 (*n* = 248, *p* = 0.0003) and TNF (*n* = 213, *p* = 0.0002) response, respectively (**Figures 5B–D**; **Supplementary Table S1**).

For the second FTI-QTL analysis, 278 participants received a BCG vaccination, representing an *in-vivo* training stimulus. Blood samples were collected before and three months after BCG vaccination. PBMCs were isolated and *ex vivo* stimulated with heat-killed *Staphylococcus aureus* (HKSA), and after 24h of incubation, TNF, IL-6, and IL-1β concentrations were measured in the supernatant. The difference in cytokine production before and 3 months after vaccination quantified the trained immune response. Seven SNPs (within a 250-kb window) in the proximity of the C1QA, C1QB, and C1QC genes were associated with the capacity of *in-vivo* BCG vaccination to induce trained immunity. Specifically, SNPs rs79073090 and rs209692 were linked to the TNF response (*n* = 273, *p* = 0.0099, *n* = 259, *p* = 0.0114). The SNP rs61767280 was associated with the IL-6 response (*n* = 273, *p* = 0.0332). SNPs rs61767328 and rs10917276 were associated with the IL-1β response (*n* = 260, *p* = 0.0108, *n* = 273, *p* = 0.0068; **Figures 5E, F**; **Supplementary Table S2**). Together, these data strengthen the hypothesis that C1q affects innate immune memory.

## 4 Discussion

We investigated the interplay between complement proteins and the immunological memory of innate immune cells. Our results demonstrated that certain complement factors can induce trained immunity, while C1q stood out as the only factor that can induce innate immune tolerance. We chose to focus on unraveling the previously unexplored regulatory role of C1q in innate immune tolerance, given its robust and consistent effects among the complement proteins we tested and its role in the pathophysiology of immune-mediated diseases. Our transcriptome and metabolic analyses findings demonstrate that the C1q-induced innate immune tolerance is accompanied by discernible changes in the metabolism of monocytes, notably a downregulation of glycolysis and mTOR signaling. Beyond metabolic shifts, we uncover concomitant changes in the epigenetic landscape, marked by an enrichment of genes showing H3K4me3 changes that resemble the previously described profile of LPS-tolerized monocytes. Validating our observations, an FTI-QTL analysis in participants from the Human Functional Genomics Project establishes a genetic association between SNPs near C1q-encoding genes (C1QA, C1QB, and C1QC) and the cytokine response associated with trained immunity in healthy adults. Our exploration reveals the C1q-mediated regulation of innate immune memory and offers new insights into the complex interaction between complement proteins and the innate immune system.

C1q is known to have anti-inflammatory properties by promoting IL-10 production, reducing IL-1α, IL-1β, IL-12, and IL-23 production by monocyte-derived macrophages, thereby downregulating Th1 and Th17 responses and inducing T-regulatory cells (40, 41). The importance of C1q in mediating immune tolerance is illustrated by a rare immune dysregulation where mutations in one of the C1q genes lead to complete C1q deficiency or low C1q levels. C1q deficiency is strongly associated with loss of immunologic tolerance, causing systemic lupus erythematosus (SLE) (42). C1q’s effect on innate immune memory may contribute to this immunological tolerance.

Our study demonstrates that C1q induces significant changes in monocyte metabolism, particularly a reduction in glycolysis. This was corroborated by our transcriptomic data, which similarly showed a suppression of glycolysis and mTORC1 signaling. Metabolic reprogramming and modulation of mTOR signaling play a central role in regulating innate immune memory. Cheng et al. demonstrated that mTOR activity, via the dectin-1-AKT-hypoxia-inducible factor 1α (HIF-1α) pathway, is elevated in trained monocytes (37). mTOR serves as a sensor of cellular energy levels, activating transcription factors that promote glycolysis and mitochondrial oxidative metabolism. In line with inhibition of mTOR and glycolysis, rapamycin limits trained immunity (37, 43). Our data showing that C1q inhibits mTOR signaling and glycolysis suggest that trained immunity plays a crucial role in the tolerizing effect of C1q.

We found that C1q-induced metabolic changes are accompanied by an increase of H3K4me3 marks in genes related to immune regulation. We compared these histone modifications with the LPS-induced histone modifications previously described by Novakovic et al. (39). Novakovic et al. described that LPS-tolerized genes are characterized by enrichment with HIF1A and TP53 motifs, and they identified p53 as a key tolerated pathway (39). This shows a significant similarity with our data, where we also found prominent H3K4me3 marks in HIF1A and TP53, among other transcription factors. The similarity suggests that the immunomodulatory effects of C1q mechanistically resemble LPS-induced tolerance.

Innate immune tolerance induced by C1q holds intriguing therapeutic potential for treating autoimmune diseases such as SLE, where C1q plays a crucial role (42). In SLE, modulating the immune response to reduce autoimmunity could potentially alleviate symptoms and prevent disease flares. Additionally, in organ transplantation, inducing immune tolerance is essential to prevent allograft rejection. By leveraging C1q’s ability to induce innate immune tolerance, it may potentially be possible to enhance the acceptance of transplanted organs, reducing the need for long-term immunosuppressive therapy and improving patient outcomes (44, 45). C1q infusion could be a therapeutic option for inducing innate immune tolerance, although a potential drawback can be that increased C1q levels could lead to easier activation of the complement system via the classical pathway. Another strategy could involve the development of agonistic molecules specifically targeting C1q receptors (C1qRs) or the development of therapeutic molecules that activate the intracellular pathways triggered by C1qRs. These molecules could mimic the effects of C1q, offering an innovative approach to treating such conditions without the need for direct C1q infusion.

Our study reveals a novel regulatory role of C1q in innate immune memory, with a tolerizing effect on the function and transcriptional profiles of monocytes. We demonstrate how C1q inhibits metabolic pathways crucial for innate immune memory. Additionally, we observed changes in C1q-stimulated monocytes resembling those seen in LPS-tolerized monocytes. Furthermore, genetic analysis links SNPs near C1q-encoding genes to cytokine responses associated with trained immunity, providing new insights into the mechanisms by which C1q promotes immunologic tolerance.

## Data Availability

The datasets presented in this study can be found in online repositories. Data will be publicly available upon publication in the GEO database. Accession numbers are GSE271218 and GSE271219 for RNA-seq and ChIP-seq data, respectively.
