# Supplementary material for: C1q reprograms innate immune memory
Source: Front Immunol. 2025 May 23;16:1515127. doi: 10.3389/fimmu.2025.1515127 (PMC12141851; doi:10.3389/fimmu.2025.1515127)
Supplement: Supplementary file 1 [file DataSheet1.pdf]

frontiers

Supplementary Material

1 Supplementary Figures and Tables

1.1 Supplementary Figures

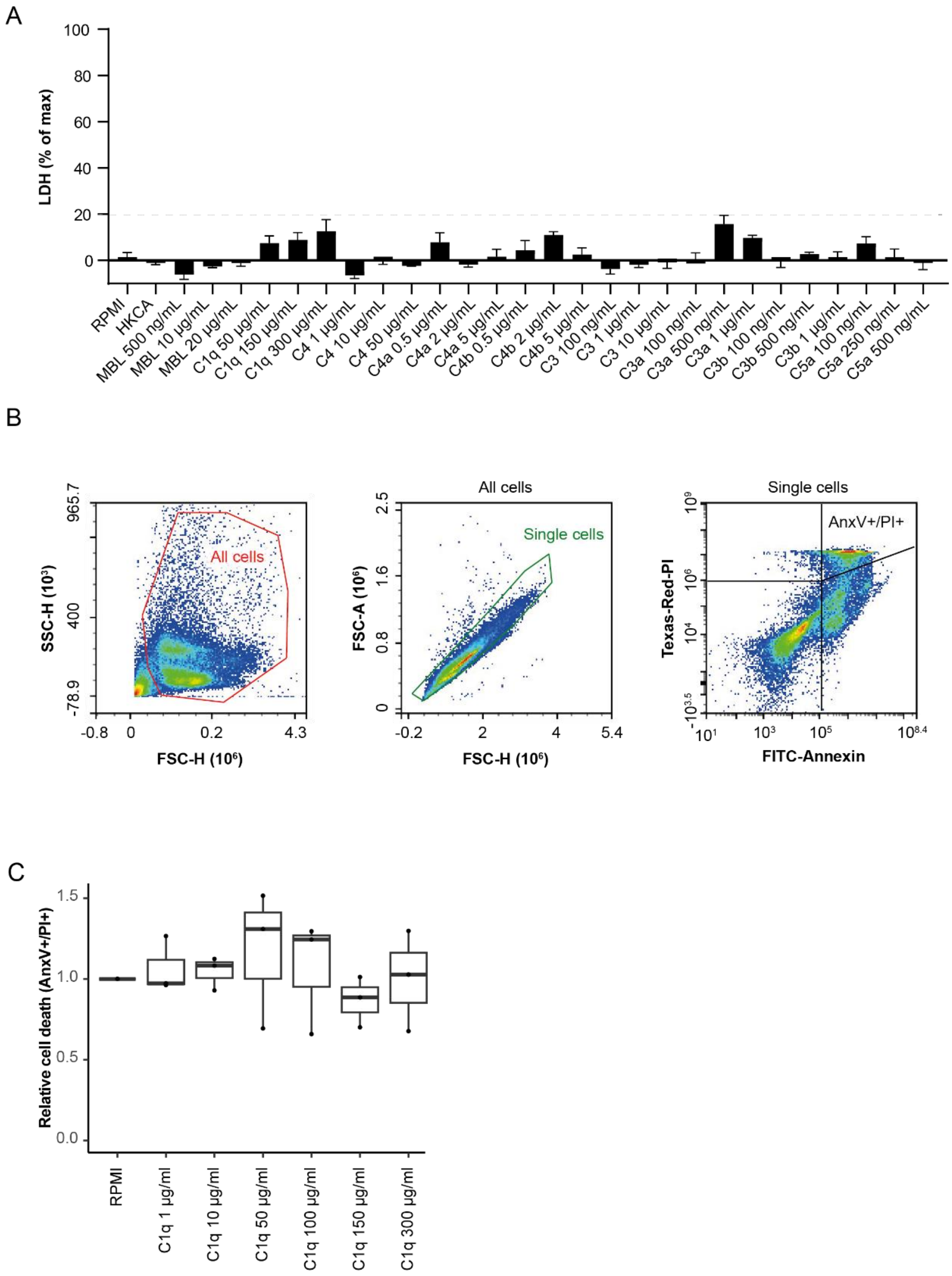

Supplementary Figure 1. Complement factors toxicity assay

(**A**) Lactate dehydrogenase (LDH) measurement of PBMCs treated with complement factors for 24 hours in three concentrations (n = 3-6 donors). (**B, C**) AnnexinV/PI stained PBMCs treated with various concentrations of C1q for 24 hours relative to negative control (RPMI) (n = 3 donors). Data are expressed as Mean ± SEM.

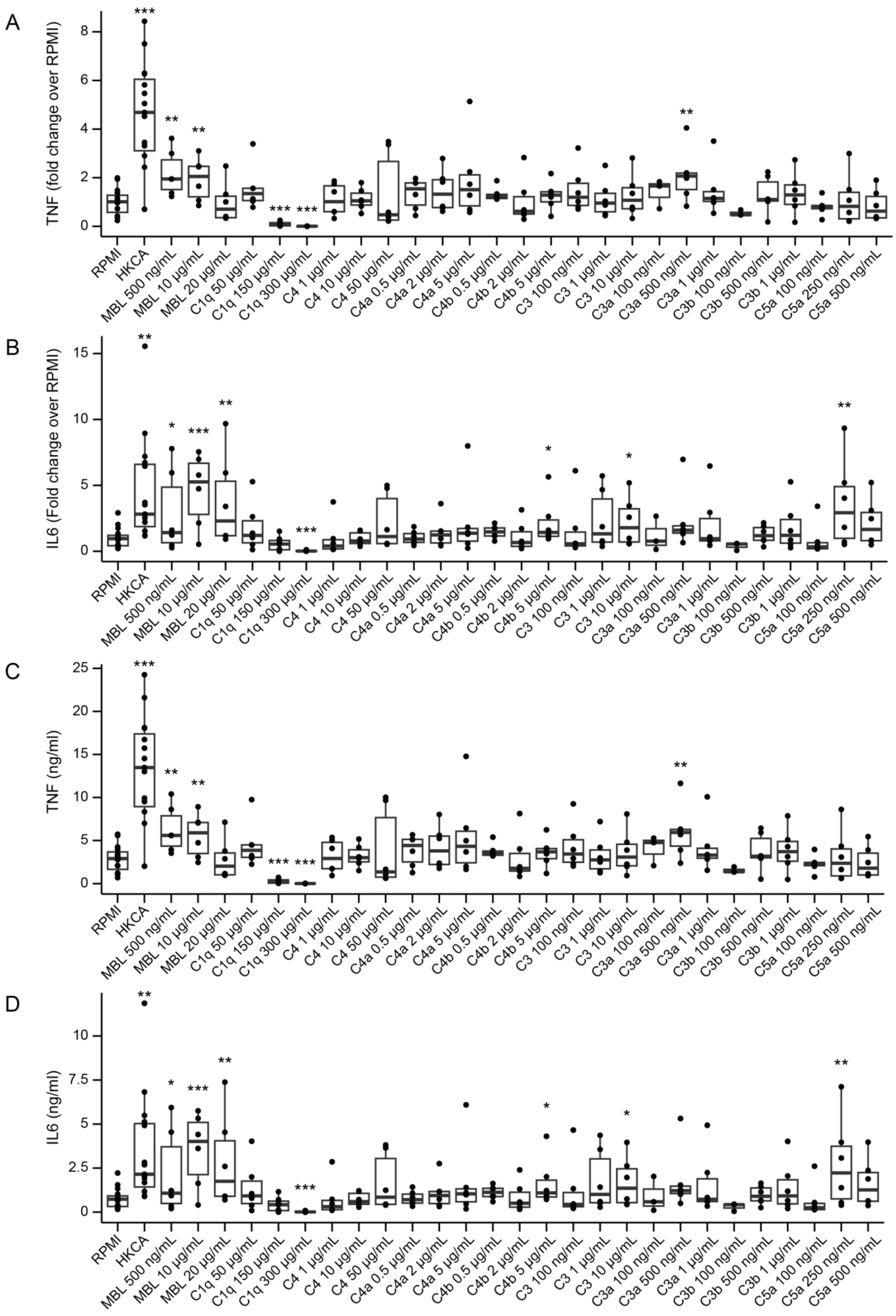
A
TNF (fold change over RPMI)
B
IL6 (Fold change over RPMI)
C
TNF (ng/ml)
D
IL6 (ng/ml)
RPMI
HKCA
MBL 500 ng/mL
MBL 10 μg/mL
MBL 20 μg/mL
C1q 50 μg/mL
C1q 150 μg/mL
C1q 300 μg/mL
C4 1 μg/mL
C4 10 μg/mL
C4 50 μg/mL
C4a 0.5 μg/mL
C4a 2 μg/mL
C4a 5 μg/mL
C4b 0.5 μg/mL
C4b 2 μg/mL
C4b 5 μg/mL
C3 100 ng/mL
C3 1 μg/mL
C3 10 μg/mL
C3a 100 ng/mL
C3a 500 ng/mL
C3a 1 μg/mL
C3b 100 ng/mL
C3b 500 ng/mL
C3b 1 μg/mL
C5a 100 ng/mL
C5a 250 ng/mL
C5a 500 ng/mL

Supplementary figure 2. Complement factors' effect on trained immunity

(**A - D**) PBMCs were stimulated for 24 hours with complement factors in three concentrations. After a five-day resting period, cells were restimulated with LPS for 24 hours, and TNF (**A, C**) and IL-6 (**B, D**) cytokine production was measured in the supernatant by ELISA (n = 12 donors for RPMI and HKCA and 3-6 donors for complement factors). Data are expressed as mean fold change compared to untrained (RPMI) PBMCs in **A** and **B** and absolute cytokine concentrations in **C** and **D**. p-values were calculated using an unpaired t-test. *$p < 0.05$, **$p < 0.01$, ***$p < 0.001$.

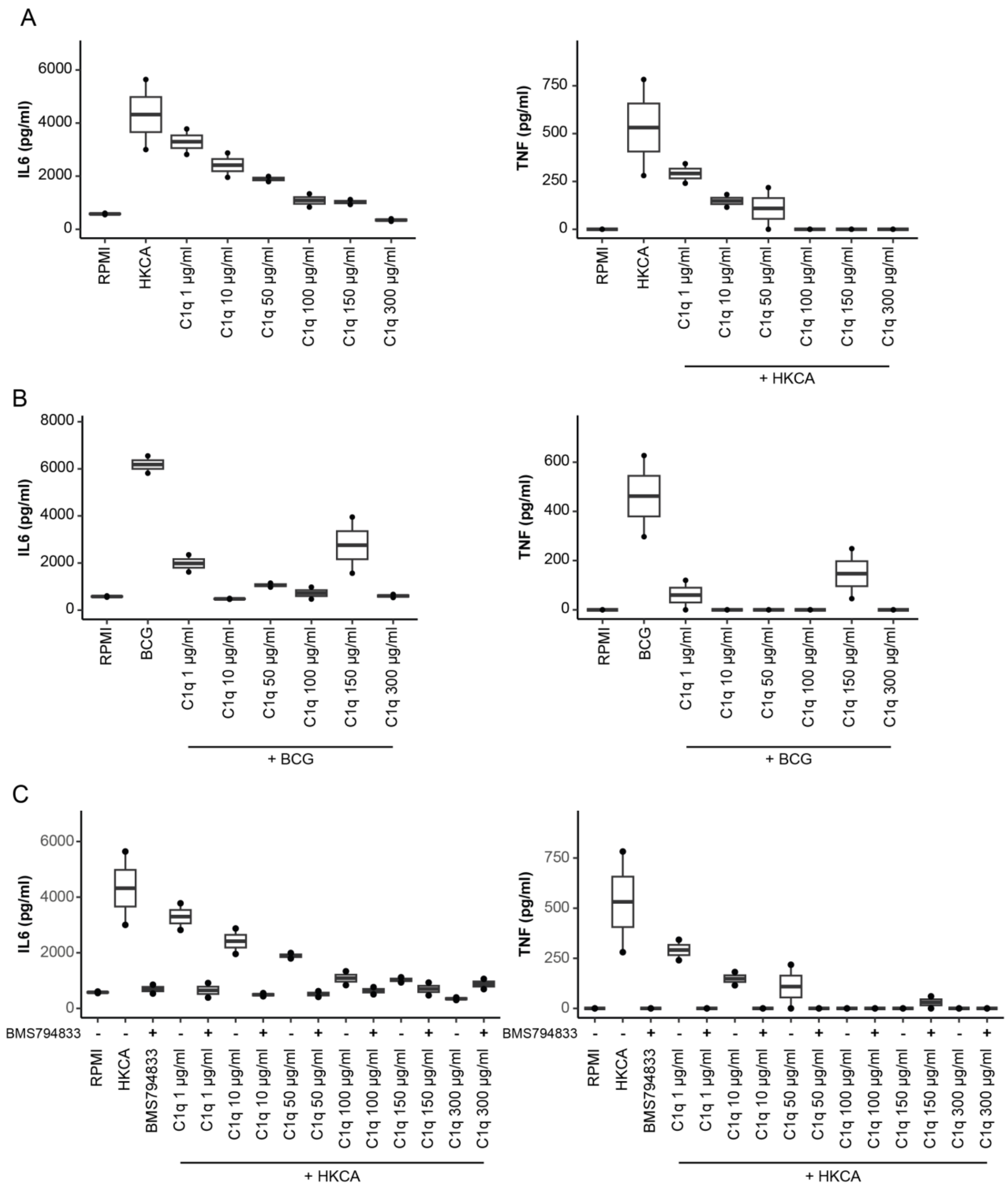

Supplementary Figure 3. C1q inhibits trained immunity induction.

(**A, B**) PBMCs were stimulated for 24 hours with HKCA (A) or BCG (B) together with various concentrations of C1q. After a five-day resting period, cells were restimulated with LPS for 24 hours, and IL-6 and TNF cytokine production was measured in the supernatant by ELISA (n = 2 donors). (C) To inhibit efferocytosis PBMCs were trained with HKCA with our without efferocytosis inhibitor BMS794833 and various concentrations of C1q (n = 2 donors).

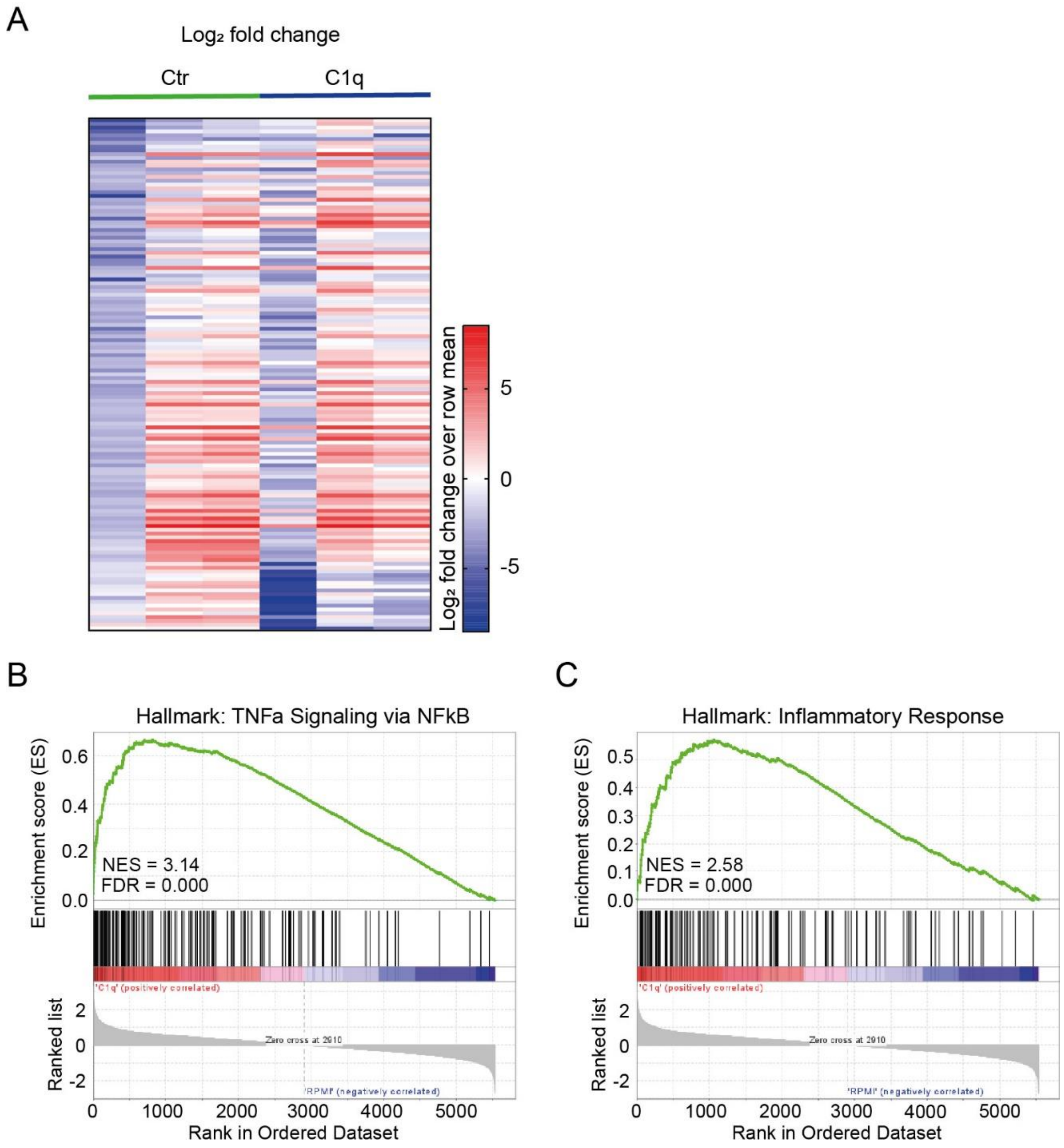

Supplementary figure 4. C1q stimulation affects the transcriptomic landscape of monocytes
(**A**). Heatmap of differentially expressed genes, p-adjusted < 0.1, fold change > 2, between monocytes stimulated with C1q (300 µg/ml) and controls (n = 4 donors).
(**B, C**). Gene set enrichment analysis of C1q (300 µg/ml) versus control (RPMI) dataset for the HALLMARK gene sets ‘TNFa Signaling via NFKB’, ‘Inflammatory Response’.
NES: Normalized enrichment score, FDR: False discovery rate.

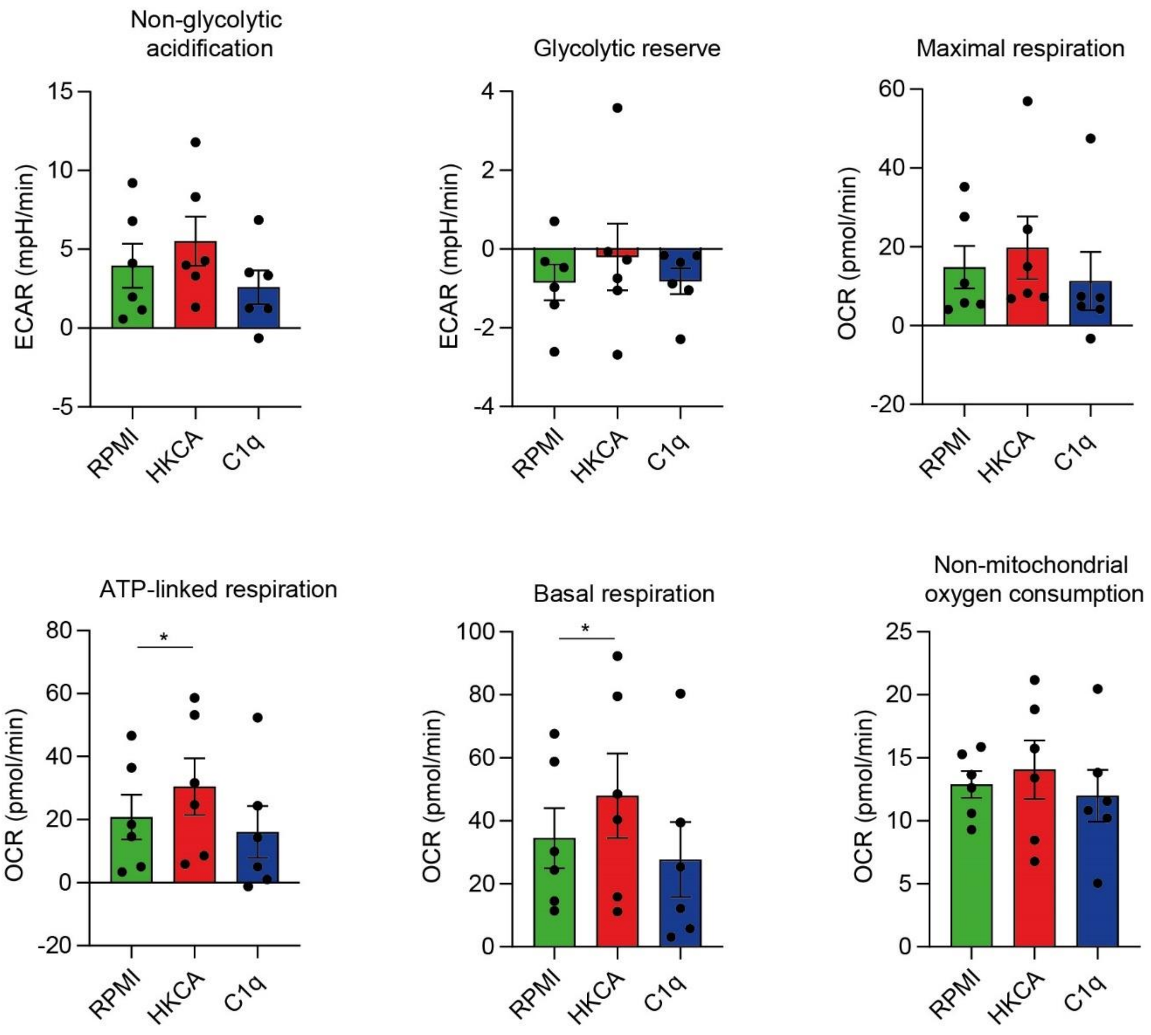

Supplementary figure 5. cell metabolism in human primary monocytes
PBMCs were stimulated for 24 hours with C1q (300 µg/ml), and with HKCA or RPMI as positive and negative controls respectively. After a five-day resting period, cellular metabolic activity was assessed by Seahorse analysis (n= 6 donors). Metabolic parameters were calculated from the oxygen consumption rate (OCR) or the extracellular acidification rate (ECAR). Data are represented as mean ± SEM. p-values were calculated using a one-tailed paired t-test.

1.2 Supplementary Tables

Supplementary table 1. FTI-QTL analysis of *in vitro* trained cytokine response

Chromosome	Chromosome start	Chromosome end	Gene	SNP	p-value	beta	ref	alt
1	22712999	23216101	C1Q	rs294179	0.0031	0.300	T	C
1	22712999	23216101	C1Q	rs666656	0.0002	0.451	G	T
1	22712999	23216101	C1Q	rs1561624	0.0030	0.605	T	G
1	22712999	23216101	C1Q	rs913243	0.0002	0.557	C	A

Supplementary table 2. FTI-QTL analysis of in vivo trained cytokine response

Chromosome	Chromosome start	Chromosome end	Gene	SNP	p-value	beta	ref	alt
1	22712999	23216101	C1Q	rs10917276	0.0068	0.142	A	G
1	22712999	23216101	C1Q	rs61767280	0.0332	-0.180	G	A
1	22712999	23216101	C1Q	rs79073090	0.0099	-0.399	G	A
1	22712999	23216101	C1Q	rs35651467	0.0112	0.669	G	A
1	22712999	23216101	C1Q	rs61767328	0.0108	-0.136	G	A
1	22712999	23216101	C1Q	rs209692	0.0114	0.233	G	A
1	22712999	23216101	C1Q	rs4578185	0.0219	-0.379	C	T
